# Symptomatic Abdominal Simple Cysts: Is Percutaneous Sclerotherapy with Hypertonic Saline and Bleomycin a Treatment Option?

**DOI:** 10.1155/2015/489363

**Published:** 2015-03-23

**Authors:** V. D. Souftas, M. Kosmidou, M. Karanikas, D. Souftas, G. Menexes, P. Prassopoulos

**Affiliations:** ^1^Department of Radiology and Medical Imaging, Medical School, Democritus University of Thrace, 681 00 Alexandroupolis, Greece; ^2^First Department of Surgery, Medical School, Democritus University of Thrace, 681 00 Alexandroupolis, Greece; ^3^Department of Social Administration, Democritus University of Thrace, 691 00 Komotini, Greece; ^4^School of Agriculture, Aristotle University of Thessaloniki, 541 24 Thessaloniki, Greece

## Abstract

*Aim.* To evaluate the feasibility of percutaneous sclerotherapy of symptomatic simple abdominal cysts, using hypertonic saline and bleomycin, as an alternative to surgery.* Materials and Methods.* This study involved fourteen consecutive patients (ten women, four men, mean age: 59.2 y) with nineteen symptomatic simple cysts (liver *n* = 14, kidney *n* = 3, and adrenal *n* = 2) treated percutaneously using a modified method. Initially CT-guided drainage was performed; the next day the integrity of the cyst/exclusion of extravasation or communications was evaluated under fluoroscopy, followed by two injections/reabsorptions of the same quantity of hypertonic NaCl 15% solution and three-time repetition of the same procedure with the addition of bleomycin. The catheter was then removed; the patients were hospitalized for 12 hours and underwent follow-ups on 1st, 3rd, 6th, and 12th months. Cyst's volumes and the reduction rate (%) were calculated in each evaluation.* Results.* No pain or complications were noted. A significant cyst's volume reduction was documented over time (*P* < 0.001). On the 12th month 17 cysts disappeared and two displayed a 98.7% and 68.9% reduction, respectively.* Conclusion.* This percutaneous approach constitutes a very promising nonsurgical alternative for patients with symptomatic simple cyst, without complications under proper precautions, leading to eliminating the majority of cysts.

## 1. Introduction

Large simple cysts in the abdomen may manifest symptoms in which case treatment is required for the patients' relief. Symptoms are nonspecific and they most commonly include discomfort, bulking symptoms, meteorism, dyspnea, nausea, vomiting, early satiety, obstructive jaundice, and upper abdominal pain, for liver cysts, discomfort, abdominal fullness, flank pain, hematuria, or hypertension in the case of renal cysts as well as eventual spontaneous or traumatic cysts' rupture [1–3].

Treatment of a simple cyst is indicated when it either becomes symptomatic or enlarges. The established treatment option is laparoscopic or/and open cyst removal. However, surgical treatment is associated with problems of morbidity and even mortality, especially in elderly patients. Additionally in the case of cysts deeply sited in the liver parenchyma or located into the posterior liver, as well as into the VII and VIII segments, it is difficult to reach them during laparoscopic exploration and they are prone to early cyst recurrence or appearance of complications [1, 2, 4–7].

The benign features of simple cysts make desirable their management with alternative less invasive methods, such as imaging guided percutaneous aspiration/drainage, as well as percutaneous drainage and sclerotherapy, often with a recurrence rate that varies and due to the presence of epithelial cell lining [8–11]. According to currently available data, percutaneous treatment for simple cysts has not been fully evaluated, yet [1].

The purpose of this study is to evaluate the feasibility of percutaneous sclerotherapy of symptomatic simple cysts, using hypertonic saline and bleomycin, as an alternative to surgery.

## 2. Materials and Methods

This prospective study involves fourteen consecutive patients (ten women, four men, mean age: 59.2 years old), with nineteen symptomatic cysts (liver *n* = 14, kidney *n* = 3, and adrenal *n* = 2) that were treated percutaneously. Patients were enrolled if they had symptoms caused by a cyst and if the cyst had the preprocedural diagnosis of “simple cyst” by imaging criteria and clinical/laboratory exclusion criteria. Infected cysts, autosomal dominant polycystic disease, and cysts that did not meet the criteria to be characterized as “simple” were excluded. Simple liver cysts were typically visualized on ultrasound images as anechoic lesions, with thin/not discrete wall, smooth borders, absence of septations, strong posterior echo enhancement, and an accentuation of echoes beyond the cyst wall. On CT/MRI scans, simple liver and adrenal cysts appeared as well demarcated lesions with homogenous fluid attenuation/intensity and without enhancement after contrast administration. The renal cysts were Bosniak category I. The preprocedural laboratory tests included liver and renal function tests and hematologic tests as well as serologic tests to exclude parasitic cysts. For adrenal cystic lesions, apart from the above mentioned imaging characteristics of “simple” cysts (endothelial or epithelial), a proper clinical and laboratory evaluation of the patients as well as biochemical and hormonal tests to exclude functioning lesions (especially pheochromocytoma) was performed [24 h urinary metanephrines (or vanillyl mandelic acid (VMA)), 17-hydroxycorticosteroids, and 17-ketosteroids measurements].

The observed symptoms included discomfort (*n* = 10 patients), bulking symptoms (*n* = 8), intermittent pain (*n* = 6), and abdominal fullness and flank pain in three patients (Table 1). When there was uncertainty about the relation between the cyst and clinical symptoms, the possibility of a pathologic condition was clinically investigated and excluded. A written informed consent was obtained from each patient and this study was approved by the ethics committee of our hospital.

The procedure was performed on an inpatient basis. The patients were hospitalized for two days. Preparation before the intervention included platelet count, prothrombin time, activating partial thromboplastin time, and approximating the international normalized ratio. Prophylactic antibiotics were used in all patients.

All patients were monitored during the procedures. Initially a CT-guided percutaneous puncture was performed, with proper selection of the entry site. The puncture site was selected so that the liver cysts are approached through the thickest possible normal liver tissue and the kidney's cysts are approached through a normal cortical tissue at Broedel's relatively “avascular” zone, if possible, in order to reduce the risk of leakage from the cyst. After determination of the puncture site, antiseptic preparation and local anesthesia with lidocaine hydrochloride 2% (Xylocaine, AstraZeneca, Rueil-Malmaison Cedex, France) were performed and a small puncture wound was made on the skin. The cyst was punctured with an 18-gauge percutaneous aspiration needle (William Cook Europe, Bjaeverskov, Denmark) and an amount of 50 mL of the cyst's contain was aspirated. The sample of the cystic fluid was sent for cytological, microbiological, and biochemical examination. A “J' shaped 0.035” guidewire was inserted into the cyst, 7-French dilatation of the percutaneous tract and an 8-French single pig-tail catheter were inserted over the guidewire. The cyst was drained by the gravity into a sac for 24 hours. The total amount of the drained fluid served as the indicator of the cysts' volume.

On the next day, the integrity of the cyst was documented fluoroscopically by injecting 50 mL of iodinated contrast medium (iopromide, Ultravist 370 mg I/mL, Bayer HealthCare Pharmaceuticals Inc., Leverkusen, Germany), dissolved in physiological sodium chloride solution (in a quantity 20–30% of the initial cyst's volume), and mixed with local anesthetic ropivacaine hydrochloride 10 mg/mL, 100 mg (Naropeine, AstraZeneca, Rueil-Malmaison Cedex, France). Possible extravasation or communications of the cysts with the biliary tree, the collecting system of the kidneys or vessels, were also precluded by the cystography under fluoroscopic control. The presence of extravasation or communication was an important exclusion criterion and no sclerotherapy was performed in these patients. This dissolution remained for 5 min in the cyst. Subsequently, after the reabsorption of the above fluid, two injections and reabsorptions of the same quantity (20–25% of the cyst's volume before intervention) of hypertonic NaCl 15% solution were effectuated, followed by three-time repetition of the same procedure with the addition of bleomycin hydrochloride for injection (Nippon Kayaku Co., Ltd., Chiyoda-ku, Tokyo, Japan) in the solution. The total dose of bleomycin administered to each of the patients was calculated on the basis of the body surface (100 mg/m^2^). A three- to five-minute time of exposure of the cyst's wall to the sclerosants before the reabsorption was considered to be crucial for the adequate contact of the entire cyst's endothelium on them. After the last reabsorption of the sclerosants, the drainage catheter was removed. The patients remained hospitalized for an additional 12 hours. Ultrasonographic (US) control and clinical evaluation, regarding the primary symptoms due to the cyst, were performed during the 12-hour postsclerotherapy hospitalization.

Clinical and imaging (US or/and CT) follow-up of the patients was performed on the 1st, 3rd, 6th, and 12th month. The cysts' volumes and the percentage of reduction rate were calculated in each evaluation. The volumes of the cysts were calculated from images, before the treatment and during follow-up examinations. Each volume was determined by measuring the spherical lesions as volume = 4/3*πr* ^3^ (where *r* is the radius of the sphere) and the nonspherical with the following equation for an ellipse: volume = length × width × height × 0.5233. The percentage of reduction rate in cyst's size was determined by calculating the difference in the volume of the cyst before and after the sclerotherapy-intervention, divided by the volume before the intervention and multiplied by 100 (so as to have a percentage) as depicted in the following equation:

(1) Cyst Volume before sclerotherapyk−Cyst Volume after sclerotherapy ·Cyst Volume after sclerotherapy−1 ×100.

The terminal goal of the treatment was the disappearance of the cyst. The cyst is considered to have disappeared if it could no longer be depicted on Ultrasonography (or Computed Tomography scans) or (on Ultrasonography) if an echogenic area was visualized in the anatomic area of the cysts' location. The disappearance of the cyst was classified as a complete regression, when the volume reduction rate was greater than 85% as a near-complete regression, when the volume reduction rate was between 50% and 85% as a partial regression and when volume reduction rate was less than 50% as no response.

All statistical analyses were performed with the SPSS v.20 software enhanced with the module “Exact Tests.” Descriptive statistical indices (minimum, median, and maximum values) were computed in order to summarize volume and reduction rate data. Following a statistically significant omnibus Friedman's test, at significance level *a* = 0.05, the cyst's sizes (volume) distributions across five time points were compared pairwise with Wilcoxon's test. The significance level in all pairwise comparisons was adjusted according to Bonferroni's criterion and was set to *a* = 0.005. In all hypotheses testing procedures, the observed significance level (*P* value) was estimated with the Exact Method (Mehta and Patel, 1999) [12]. Using this method the inferential decisions are valid even in cases where the methodological presuppositions of the corresponding nonparametric tests are not satisfied.

## 3. Results

None of the patients has complained from being in pain during sclerotherapy. No intervention-related immediate complications, such as vasovagal reflex, bleeding, fever, infection, flu-like symptoms, hair loss, target-organ dysfunction, or damage to adjacent organs, were observed.

Eight patients claimed symptoms relief even during the first hours after the treatment. For the remaining six patients complete relief of the symptoms was achieved 1-2 hours after the treatment of the second (four patients) or the third cyst (one patient).

No abnormalities were detected in the bacteriological, parasitic, cytological, or biochemical studies of the cystic fluid in all cases.

There was no evidence of procedure-related disease during the follow-up period. Neither major nor minor complications related to the procedure were encountered in short or long term follow-ups, especially regarding the use of the sclerosing agents (pain, other local or general symptoms indicative of tissue irritation, flu-like symptoms, cholangitis, cholangiofibrosis, interstitial pulmonary fibrosis, or skin hyperpigmentation).

**Figure 1:**
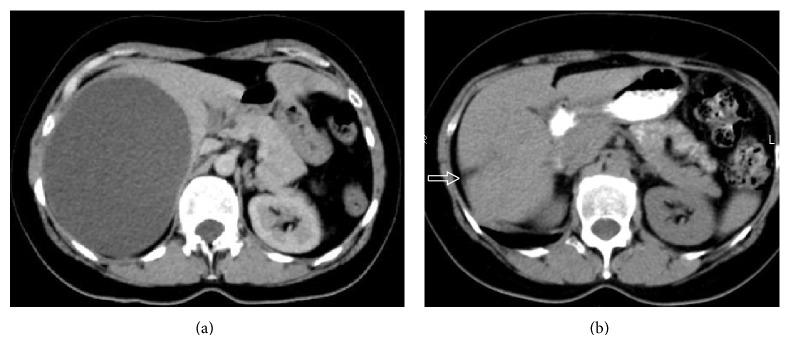
Axial Computed Tomography scans of a 48-year-old Caucasian woman with a large simple cyst located at segment IV of the liver [cyst number 3], before (a) and 6 months after percutaneous sclerotherapy with combination of hypertonic NaCl 15% and bleomycin (b). After treatment [image b], only a simple scar is obvious in the liver parenchyma [arrow].

**Figure 2:**
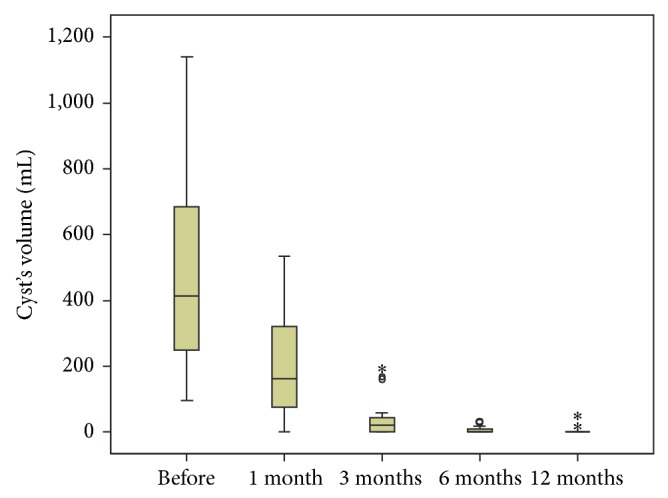
The cysts' volume distribution, before treatment and after one, three, six, and twelve months after treatment. ○ and ∗ denote outliers.

A significant decrease of the cysts' volume was noted at follow-ups. Cysts size decreased gradually over the follow-up period (Figure 1).  Table 2 shows the calculated cysts' volumes before treatment and at follow-up.  Figure 2 (box plot) shows the cysts' volume distribution, before and in the 1st, 3rd, 6th, and 12th months after treatment. A less optimal result was noted on the smallest of three cysts (14, 15, and 16, Tables 1 and 2) treated in one session. Only two of them (14 & 15) were completely resolved despite cyst number 16 being located at liver segment VI.

**Figure 3:**
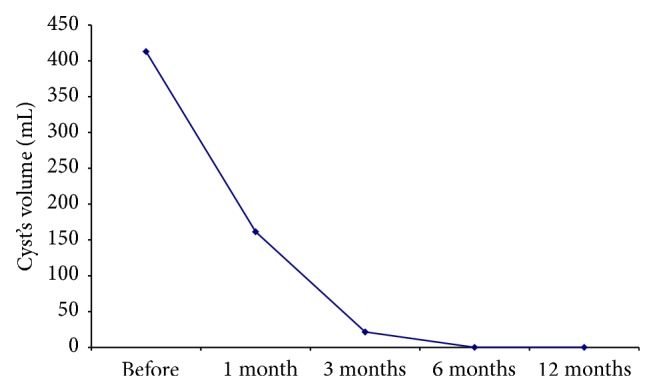
The median initial cysts volume (mL) and its reduction after one, three, six, and twelve months after treatment.

Data analysis (Table 3) indicates that there was a statistically significant volume reduction over time (Friedman's test *P* < 0.001). The median reduction rate of the cyst's volume was 58% in the 1st month, 92.6% in the 3rd month, and 100% in the 6th and 12th months (Figure 3). At the 12th month follow-up 17 cysts disappeared (complete regression) and 2 displayed a reduction rate of 98.7% (near-complete regression) and 68.9% (partial regression), respectively. Volume at month 6 is not statistically different from volume at month 12 (*P* > 0.005).

## 4. Discussion

Therapeutic consequences can only be driven if the cysts become symptomatic because of their position, increasing size, hemorrhage, or superinfection. Τhere has been no consensus on the optimal approach to management of simple cystic disease [13].

Conventional surgical treatment of simple hepatic cysts consisted of complete excision of the cyst. However, the high incidence of complications associated with such a radical procedure led to the conclusion that deroofing the cyst (“fenestration,” “marsupialisation”) was an effective mode of treatment [14, 15]. The technique during the laparoscopic procedure is also important for the outcome [2, 7, 13]. Fenestration of the cyst by laparotomy or laparoscopic deroofing, with the widest possible excision of the wall and coagulation, showed high success rates and was regarded as the standard procedure for a long time [13]. Lin procedure by deroofing the cyst wall using electrocautery or harmonic shears is important to avoid cyst recurrence, but great precautions should be taken to keep a 1 cm distance from the parenchymal liver edge in order to avoid bleeding or bile leak from liver parenchyma [7]. When deroofing, special caution should be taken not to resect hepatic parenchyma, given that transected bile ducts may lead to postoperative bile leaks [2]. Reported complications associated with laparoscopic treatment of simple liver cysts include wound infection, bile leak, chest infection, subphrenic hematoma, and prolonged drainage after procedure [16].

Laparoscopic treatment is now the golden standard for treating selected, huge, accessible, highly symptomatic, or complicated liver cysts. In contrast, the laparoscopic approach is not useful for patients suffering from adult polycystic liver disease (PLD), except for type I PLD with large multiple hepatic cysts [13–15]. A strict selection of patients is mandatory. The best candidates for a laparoscopic approach are large, superficial, accessible cysts at the liver surface, located in the anterior segments of the right liver or in the lateral left liver (segments II to VI in the Couinaud classification) [13]. Large cysts in the right posterior lobe have a high recurrence rate [2]. Atypical hepatic resections are the best technique to treat large, simple hepatic cysts, especially those located in the intermediate and posterior segments [17]. Simple cysts located in segment VIII are more prone to early cyst recurrence after laparoscopic deroofing because the residual cyst cavity is immediately covert by the diaphragm, except if in situ omentoplasty is employed to obliterate the cystic cavity [6].

A few results of surgical treatment for simple liver cysts were reported. Laparoscopic management of simple cystic lesions of the liverhas complications for up to 18% of the cases, procedure's conversion for up to 23%, reoperation for up to 18%, and recurrence rate for up to 44%. The worldwide reported experience with laparoscopic management of simple liver cysts varies in different series and is detailed in Table 4 [5, 6, 18–24].

The Bosniak renal cyst classification has, thus far, passed the test of time and has been a useful method for diagnosing and suggesting the management of cystic lesions of the kidneys [25, 26]. Although a causative association between simple renal cysts and patient symptoms is not always justified, the vast majority of studies indicate that symptomatic renal cysts should be treated. Symptomatic simple cysts can be treated in various ways, ranging from simple aspiration, with or without the use of sclerotic agents, to surgical excision via open, percutaneous, laparoscopic, or robotic surgery [27, 28]. Laparoscopic deroofing treatment for simple renal cysts seems to be more effective than percutaneous methods, but it needs hospitalization for a significantly longer period, while complications are happening too [29]. Randomised studies with large patient groups are required to compare effectiveness, complications, and costs of laparoscopic and percutaneous techniques [27]. Up to date, percutaneous drainage and sclerotherapy for symptomatic simple (Bosniak category I) renal cysts have been used as a frontline treatment before surgical and laparoscopic methods because of their minimally invasive nature [30–40].

Adrenal cysts are rare and are pathologically classified as endothelial, epithelial, pseudocystic, or parasitic. Adrenal endothelial cysts are the most common ones, followed by pseudocysts [41]. Endothelial cysts comprise 45% of adrenal cysts. Imaging usually describes endothelial cysts the same as seen for simple cysts of other organs. Epithelial cysts or “true cysts” are much like “simple” endothelial cysts, with smooth, flattened lining, albeit their walls are lined with true epithelium. They are rare, with 6–9% incidence, and some authors doubt their existence because adrenal cells do not contain any true acini from which a follicular cyst may develop [42]. Potential interventions include percutaneous needle aspiration or sclerotherapy, surgical resection, or cyst unroofing. Percutaneous management has been suggested as an alternative treatment option if the cyst is not hormonally active and if there is no suspicion of malignancy [43, 44]. Surgical excision is indicated in the presence of symptoms, suspicion of malignancy, and increase in the size or detection of a functioning adrenal cyst. En bloc adrenalectomy, preferably by a laparoscopic approach, has become the treatment of choice [45, 46].

Percutaneous aspiration of hepatic cysts does not provide definitive therapy and has a high recurrence rate (78–100%) due to the presence of epithelial cell lining [8–10]. Mazza et al. agree that recurrence after simple puncture and aspiration of the cyst is almost guaranteed [2]. High rate of recurrence after treatment of the cysts only by percutaneous drainage is data consistent with other reports [1, 47, 48]. Percutaneous drainage, with or without sclerosing agents, is the most preferable among the alternative methods of simple cysts' treatment [4, 10, 30]. Several studies evaluated the effectiveness of percutaneous drainage using various sclerosants in the treatment of abdominal cysts [4]. Several sclerosing agents have been used, including ethanol, glucose, phenol, iophendylate, pantopaque, minocycline hydrochloride, povidone-iodine, n-butyl cyanoacrylate, holmium-166-chitosan complex, ethanolamine oleate, tetracycline hydrochlorate, doxycycline, hypertonic saline solution, and bleomycin [3, 31, 49–52]. The sclerosing agent mostly used is ethanol [1, 4, 33–36, 47, 48]. The treatment with alcohol varies significantly in various studies with respect to the time of exposure to ethanol and the number of sclerotherapy sessions [1]. Egilmez et al. [3] concluded that single-session and multiple sessions using ethanol sclerotherapy are equally effective procedures, with or without very low rate of recurrence (2%). Alcohol destroys the cells lining into the cyst cavity, thus disabling cystic fluid secretion and resulting in cyst resolution [9]. The mechanism of alcohol sclerotherapy involves protein denaturation, cell death, and fibrous scarring. However the main disadvantage of this method is the remarkably intense pain induced during the cyst's filling [1].

Only two studies were found in the literature referring to the evaluation of the percutaneous use of hypertonic saline for the management of simple renal cysts [3, 50], while there are several published studies related to the successful use of hypertonic saline sclerotherapy in patients with hydatid liver cysts as a primary treatment [3]. According to Egilmez et al., sclerotherapy with 95% ethanol is more effective and more painful than 20% hypertonic saline sclerotherapy [3].

Bleomycin sulfate for injection is a mixture of cytotoxic glycopeptide antibiotics isolated from a strain of streptococcus verticillus, useful in the management of squamous cell carcinoma, testicular carcinoma, and lymphomas. It has also been proven to be an effective sclerosant in the treatment of malignant pleural effusion and recurrence of pleural effusion [53, 54]. Duncan and van der Nest proposed bleomycin intralesional injection as an alternative to the treatment of recurrent intractable epistaxis in patients with hereditary hemorrhagic telangiectasia [54]. The induction of sclerosis is mediated by inflammatory and fibrogenic cytokines as well as by the direct effect of bleomycin on extracellular matrix synthesis in fibroblasts [55]. Percutaneous intralesional administration of bleomycin, as sclerosant, to treat lymphatic and slow-flow vascular malformations, is in use for many years [56, 57]. Single-session percutaneous needle aspiration and single-injection bleomycin sclerotherapy have been recently used for the management of simple renal cysts [51].

Table 5 shows that percutaneous treatment of symptomatic simple cysts of the liver and kidneys, using sclerosants or prolonged catheter drainage with negative pressure, is effective and safe [1, 9, 30–32, 47, 50–52, 58–65]. The complications noted were minor, such as pain, vasovagal reflex, fever, nausea, causalgia, drunkenness (if alcohol was used), and very rarely complications that need surgical treatment. The use of minocycline hydrochloride as a sclerosant, with sessions repeated daily for 7-8 days, performed in a limited number of patients, seems to be the most effective one [65]. Prolonged catheter drainage with negative pressure for 24 hours seems to be as effective as alcohol sclerotherapy [1, 56]. In the latter, alcohol has to be retained into the cyst for several times (20 min to 4 hours) triggering several complications during the procedure, mostly pain [1, 30, 47, 56, 58–61, 63].

In this study for the first time combination of hypertonic saline 15% and bleomycin as sclerosants is used for percutaneous treatment of simple cysts. This combination proved to be very effective in our cases, since 17/19 cysts (81%) disappeared and the remaining two displayed a reduction rate of 98.74% (near-complete response) and 68.9% (partial regression), respectively. These data promise a lot and are of the best, compared with those published by others for percutaneous treatment of simple cysts at any location (Table 5). An important advance of this method is the absence of pain during the treatment. This is attributed to the local anesthetic, which we introduced into the lumen of the cyst, mixed with the iodinated contrast medium to sustain the cysts' integrity, before the sclerosants administration. Furthermore, cysts' integrity was fundamental for the short and long term patient's safety, when agents as hypertonic saline and bleomycin were administered.

In conclusion, the results of our study suggest that percutaneous sclerotherapy using hypertonic NaCl 15% and bleomycin is a very promising nonsurgical alternative method for patients with symptomatic simple cyst leading to elimination in the majority of cysts. The proposed technique is a one-session intervention that can be done with very short hospitalization (or even in outpatient basis), not painful at all, and without complications when performed under the proper precautions. Larger scale studies are needed to provide the value of percutaneous sclerotherapy using hypertonic sodium chloride and bleomycin regarding the treatment of the simple cysts at any location.

## Figures and Tables

**Table 1:** Patients' data and cysts' location, symptomatology, and initial volume.

Patient number	Cyst number	Age/sex	Location	Symptoms	Initial cyst's volume
35277	1	47/female	Left kidney	Abdominal fullness, flank pain	486 mL
39401	2	76/female	Liver/segment IV	Discomfort, bulking symptoms	291.25 mL
34958	3	48/female	Liver/segment VI	Discomfort, intermittent pain, and bulking symptoms	670.8 mL
37154	4	57/male	Liver/segment III	Discomfort, intermittent pain, and bulking symptoms	1141.2 mL
23372	5	56/male	Right adrenal	Abdominal upper quadrant pain	321.4 mL
36237	6	60/female	Liver/segment VII	Discomfort, bulking symptoms	585.2 mL
36237	7	60/female	Liver/segment VIII	Discomfort, bulking symptoms	403.75 mL
33217	8	52/male	Right adrenal	Right abdominal upper quadrant pain	904.32 mL
42847	9	52/female	Liver/segment VIII	Discomfort, bulking symptoms	412.87 mL
42711	10	42/male	Right kidney	Abdominal fullness, flank pain	220.78 mL
42711	11	42/male	Right kidney	Abdominal fullness, flank pain	241.4 mL
28254	12	71/female	Liver/segment VI	Discomfort	253.43 mL
26097	13	77/female	Liver/segment V	Discomfort, intermittent pain	696.56 mL
27430	14	76/female	Liver/segment IV	Discomfort, intermittent pain	514.1 mL
27430	15	76/female	Liver/segment VI	Discomfort, intermittent pain	201.6 mL
27430	16	76/female	Liver/segment VI	Discomfort, intermittent pain	94.5 mL
29977	17	57/female	Liver/segment VI	Discomfort, bulking symptoms, and intermittent pain	1073.36 mL
29977	18	57/female	Liver/segment IV	Discomfort, bulking symptoms, and intermittent pain	234.6 mL
37482	19	58/female	Liver/segment III	Discomfort	388.126 mL

**Table 2:** Cysts' volume/diameter before and after treatment (at 1st, 3rd, 6th, and 12th months).

	Initial cysts' size		After 1st month size		After 3rd month size		After 6th month size		After 12th month size	
	Volume (mL)	Diameter (cm)	Volume (mL)	Diameter (cm)	Volume (mL)	Diameter (cm)	Volume (mL)	Diameter (cm)	Volume (mL)	Diameter (cm)
1	486.00	9.8	161.35	6.8	44.58	4.4	6.32	2.3	0.00	0.0
2	291.25	8.2	157.40	6.7	21.56	3.5	13.40	2.9	9.20	2.6
3	670.80	10.9	109.76	5.9	0.00	0.0	0.00	0.0	0.00	0.0
4	1141.20	13.0	465.75	9.6	0.00	0.0	0.00	0.0	0.00	0.0
5	321.40	8.5	49.04	4.5	30.34	3.9	2.08	1.6	0.00	0.0
6	585.20	10.4	347.50	8.7	45.60	4.4	1.34	1.4	0.00	0.0
7	403.75	9.2	236.20	7.7	33.11	4.0	4.68	2.1	0.00	0.0
8	904.32	12.0	533.55	10.1	0.00	0.0	0.00	0.0	0.00	0.0
9	412.87	9.2	223.13	7.5	168.45	6.9	0.00	0.0	0.00	0.0
10	245.65	7.8	81.50	5.4	8.93	2.6	0.00	0.0	0.00	0.0
11	195.33	7.2	3.00	1.8	0.00	0.0	0.00	0.0	0.00	0.0
12	253.43	7.9	87.12	5.5	0.00	0.0	0.00	0.0	0.00	0.0
13	696.56	11.0	378.93	9.0	57.12	4.8	29.60	3.8	0.00	0.0
14	514.10	9.9	295.10	8.3	188.96	7.1	13.50	3.0	0.00	0.0
15	201.60	7.3	171.40	6.9	159.98	6.7	18.00	3.3	0.00	0.0
16	94.50	5.7	49.90	4.6	36.54	4.1	32.50	4.0	29.40	3.8
17	1073.30	12.7	450.81	9.5	0.00	0.0	0.00	0.0	0.00	0.0
18	234.60	7.7	0.00	0.0	0.00	0.0	0.00	0.0	0.00	0.0
19	388.13	9.1	184.34	7.1	44.20	4.4	0.00	0.0	0.00	0.0

**Table 3:** Cysts' volume reduction over time after treatment.

	Before	Month_1	Month_3	Month_6	Month_12
Minimum volume (mL)	94.48	0	0	0	0
Median volume (mL)	412.87^**^	161.35^**^	21.55^**^	0^**^	0^**^
Maximum volume (mL)	1,141.19	533.55	188.96	32.48	29.40
Friedman's test	*P* < 0.001				
Minimum reduction rate (mL)		15.0%	20.6%	65.6%	68.9%
Median reduction rate (mL)		58.0%	92.6%	100.0%	100.0%
Maximum reduction rate (mL)		100.0%	100.0%	100.0%	100.0%

^**^Difference in the median volume of the cysts is statistically significant at a *P* < 0.005 level according to the results of a series of Wilcoxon's tests.

**Table 4:** Reported laparoscopic management of simple cystic lesions of the liver.

Authors	Conversion	Complications	Reoperation	Follow-up	Recurrence
Gigot et al. [5]	6%	15%	6%	1–48	44%
Katkhouda et al. [18]	6%	6%	6%	3–78	0%
Payatakes et al. [19]	0	17%	8%	4–84	17%
Zalaba et al. [20]	0	0	9.5%	1–54	0%
Zacherl et al. [21]	0	9%	18%	6–76	14.3%
Gigot et al. [6]	23%	18%	6%	3–122	0%
Fiamingo et al. [22]	0	10%	0	34	10%
Kwon et al. [23]	0	0	0	10–87	0%
Gall et al. [24]	0	15%	2	19	4%

**Table 5:** Reported percutaneous treatment of simple cystic lesions.

Authors	Drainage catheter/ sclerosant used	Organ of cysts' location	Number of sessions/duration of follow-up	Complications/ percentage	Complete regression (CR) at one year (disappearance of the cyst, after the first session)	Near-CR at one year (volume reduction rate, after the first session >85%)	Partial regression at one year (volume reduction rate, after the first session 50–85%)	No response at one year (volume reduction rate <50%)
Saini et al. [9]	No (aspiration, only)/none	Liver	One or two/up to 24 mos.	—	0%	0%	0%	100%

^*^Zerem et al. [1]	Yes/group I: prolonged catheter drainage for 24 hours Group II: ethanol, retention for two hours	Liver	One (or two in 12.5% of group I & 26.01 of group II)/24 mos.	Group I: pain/16.6% Group II: pain/30.4%, fever/13%, drunkenness/30.4%, headache/26.01%, and deep sleep/8.7%	Group I: 66.7% Group II: 47.8%	Obscure	Obscure	Obscure

Kairaluoma et al. [58]	Yes/ethanol	Liver	One or two/12 to 32 mos.	Pain, fever, and nausea-vomiting/72.7%	37.5%	—	62.5%	—

Montorsi et al. [59]	Yes/ethanol	Liver	One/6 to 60 mos.	Pain, fever/9.5%	71.4% (follow-up period 12–24 mos.)	Obscure	Obscure	28.5%

Larssen et al. [60]	Yes/ethanol	Liver	One/12 to 47 mos.	Pain/80%	30%	40%	20%	10%

^*^Yang et al. [61]	Yes^*^/ethanol group I: 4-hour retention, group II: 2-hour retention	Liver	One/9 to 59 mos.	Intractable pain (32.26%), symptoms and signs of drunkenness (80.6%), flushing and headache (54%), skin rash (9.6%), deep sleep (6.4%), and blood pressure of 30–50 mm Hg (22.56%).	16.12%	Obscure	Obscure	Obscure

Zerem et al. [62]	Yes. Two groups: continuous 24-hour catheter evacuation (I) and drainage for two hours (II)/ethanol	Kidney	One/24 mos.	Pain/44.3% [moderate in 26.09% of cases, severe in 17.4%]	40% (at 24-month follow-up, 52% of continuous group I and 28% of group II)	Obscure	Obscure	7.06%

Touloupidis et al. [30]	Yes, continuous drainage of the cyst for 24 h/ethanol, retention for 20 min	Kidney	One or multiple (in most of cases)/5 years	Pain/obscure, purulence of the cyst that required open surgery/0.4%	0	7.0%	22.0%	Obscure

Lin et al. [63]	Yes/ethanol group I: 4-hour retention, group II: 2-hour retention	Kidney	One/12 mos. (at least)	Pain (most mild to moderate, severe 13.9%), nausea, causalgia, drunkenness, perforation to the collecting system (2.7%)	Group I: 21.4%, group II: 52.4%	65.7%	2.8%	0

Akinci et al. [47]	Yes/ethanol	Kidney	One/12 to 85 mos.	Retroperitoneal hematoma, spontaneous hemorrhage into the cyst cavity/2%	11.2% (17.5%, during the total follow-up period)	Obscure	Obscure	Obscure

Cho et al. [64]	Yes/acetic acid (group I) and ethanol (group II)	Kidney	One/group I: 18 mos., group II: 13 mos.	Pain/group I: 48.3%, group II: 12.5	Group I: 90.6% Group II: 60%	—	Group I: 9.4% 0% Group II: 30%	—

Kwon et al. [31]	Yes/50% acetic acid (group I: sclerosant retention into cyst for 20 min, group II: retention for 5 min)	Kidney	One/group I: 12 to 52 mos., group II: 7 to 48-mos.	Pain/7.7%	Group I: 53.1% Group II: 48.57% CR, during the total follow-up period: group I: 66% group II: 63%	All the rest, during the total follow-up period Percentage of the reduction rate of the cyst's volume: group I: 97.4%% group II: 96.9%	0	0

Nakaoka et al. [49]	Yes/ethanolamine oleate	Liver (*n* = 15 PLD and *n* = 2 simple cysts)	One or two (one patient)/<1 (two patients) up to 95 mos. (median 44.4 mos.)	Pain (mild to severe), vasovagal reflex, and mild fever/64.7%	—	88.4% (total)	—	—

Yamamoto et al. [32]	Yes/ethanolamine oleate	Liver, kidney	One/4 to 10 mos.	Fever/28.5%	0	100%	0	0

Yoshida et al. [65]	Yes/minocycline hydrochloride	Liver	Session repeated daily for 7-8 days/42 to 153 mos.	Pain/22.2%	100%	0%	0%	0%

Jusufovic and Zerem [50]	Yes/20% NaCl solution	Liver	One/24 mos.	—	40.0%	55%	5%	0

Li et al. [51]	No (aspiration, only)/bleomycin	Kidney	One/12 mos.	No major	47.0%	36.4%	15.1%	1.5%

^*^2% lidocaine was injected into the cyst after evacuation and before treatment for pain relief.
